# *Allium cepa*: A Treasure of Bioactive Phytochemicals with Prospective Health Benefits

**DOI:** 10.1155/2022/4586318

**Published:** 2022-01-18

**Authors:** Arka Jyoti Chakraborty, Tanvir Mahtab Uddin, B. M. Redwan Matin Zidan, Saikat Mitra, Rajib Das, Firzan Nainu, Kuldeep Dhama, Arpita Roy, Md. Jamal Hossain, Ameer Khusro, Talha Bin Emran

**Affiliations:** ^1^Department of Pharmacy, Faculty of Pharmacy, University of Dhaka, Dhaka 1000, Bangladesh; ^2^Faculty of Pharmacy, Hasanuddin University, Tamalanrea, Kota Makassar, Sulawesi Selatan 90245, Indonesia; ^3^Division of Pathology, ICAR-Indian Veterinary Research Institute, Izatnagar, Bareilly 243122, Uttar Pradesh, India; ^4^Department of Biotechnology, School of Engineering & Technology, Sharda University, Greater Noida 201310, India; ^5^Department of Pharmacy, State University of Bangladesh, 77 Satmasjid Road, Dhanmondi, Dhaka 1205, Bangladesh; ^6^Research Department of Plant Biology and Biotechnology, Loyola College, Chennai 34, Tamil Nadu, India; ^7^Department of Pharmacy, BGC Trust University Bangladesh, Chittagong 4381, Bangladesh

## Abstract

As *Allium cepa* is one of the most important condiment plants grown and consumed all over the world, various therapeutic and pharmacological effects of *A. cepa* were reviewed. Onion (*Allium cepa*) is a high dietary fiber-rich perennial herb that is placed under the family Amaryllidaceae. It contains high concentration of folic acid, vitamin B6, magnesium, calcium, potassium, and phosphorus as well as vitamins and minerals. It is widely used as an antimicrobial agent, but it showed anticancer, antidiabetic, antioxidant, antiplatelet, antihypertensive, and antidepressant effects and neuroprotective, anti-inflammatory, and antiparasitic effects and so on. It is said to have beneficial effects on the digestive, circulatory, and respiratory systems, as well as on the immune system. This review article was devoted to discussing many health benefits and traditional uses of onions in pharmacological perspectives, as well as the safety/toxicological profile. If more detailed research on this perennial herb is conducted, it will open the door to an infinite number of possibilities.

## 1. Introduction

*Allium cepa* (also known as onion) is a perennial herb with the stem in the underground bulb. Onions belong to the Liliaceae family, while some authors mention them as Alliaceae. Common onion has one or two leafless flower stalks reaching 75–180 cm (2.5–6 feet) in height. Most commercially cultivated onions are cultivated from the thin, dark seeds of the plant. Onion is highly regarded and stored as pickles for its flavor and nutritious values. Onions are thought to have originated in Afghanistan/Iran/USSR and are now produced in more than 175 countries [1]. Onion has a high dietary fiber and sugar content of about 90 percent water. Onion has a high dietary fiber and sugar content of about 90 percent water. A diet rich in vegetables has been identified as providing a number of health benefits to preventing two of the more prevalent and relevant diseases nowadays [2]. Onions contain a number of vitamins (B2, C, and B1), selenium, and potassium. They can cure diabetes mellitus, CVDs, and stomach cancer. Onion peel reveals biological effective hypertrophic scar and keloid prevention. Studies demonstrate that onion extract is capable of removing hypertrophic wounds too [3]. Regular intake of onions lowers the risk of colorectal, lung, liver, brain, stomach, ovarian, prostate, and breast cancer [4]. The function of antiplatelets is significantly influenced by genotype, climate, and vegetable storage period. *A. cepa* (onions) have antioxidant potential due to the presence of high amounts of organosulfur compounds, polyphenols, and flavonoids which are natural antioxidants. Garlic and onion extract effectively destroys all parasites and suppresses the irreversible reductase *Trypanosoma brucei* trypanothione. Studies demonstrated that onion shows an antidepressant effect too. *A. cepa* and its constituents, especially quercetin, are possible immunomodulatory therapeutic candidates for the treatment of immune dysregulation disorders. The anti-inflammatory and protective impact of *A. cepa* on tracheal tolerance and lung inflammation in asthmatic animals means that it may be used to treat airway disorders like asthma [5–9]. In the presence of liver-damaging or liver-harmful ethanol, *A. cepa* extracts were found to have hepatoprotective effects. Aqueous extract of *A. cepa* bulb has essential antioxidant and hepatoprotective function against ethanol-induced hepatotoxicity [10]. However, researchers found some toxicity reports on onion too. Researches show that OE has an antioxidant potential that protects it from oxidative harm. Onion toxicity induces hemolytic anemia in puppies, according to the researchers [11]. As a result, the goal of this study is to conduct a thorough analysis of the existing literature on *A. cepa*'s chemical and morphological features, pharmacological properties, and therapeutic actions, as well as clinical and preclinical research.

## 2. Morphological Characteristics of *Allium cepa*

*A. cepa* is placed under the family Amaryllidaceae. It is perennial herb with a stem in underground bulb. The root system is fibrous adventitious. The underground bulb, cylindrical and flesh, with sheathy leaf foundation with a parallel venation appears from a cluster of progressive leaves. Pedicels are of the same length, derived from the peduncle apex that equals all flowers. Flowers are ebracteolate, bracteate, and hypogynous, tiny, complete, blonde, trimerous, actinomorphic, and protandrous. There are 6 tepals, arranged, white in two whorls of three each, syntepalous showing aestivation of valvate. There are 6 stamens, arranged in two whorls of three each, apostamenous, epitepalous, and opposite to tepals. Introse, basifixed, anthers dithecous, and dehiscing longitudinally. The gynoecium is syncarpous and tricarpellary. Ovary trilocular, superior with two ovules in each locule on axile placentation. The style of gynoecium is simple with slender stigma. Fruit is loculicidal capsule. Its seed is endospermous. In Asia, about 660 allium species are found. But in Central America, Africa, and South America, some species are found. Onions are thought to have originated in Afghanistan/Iran/USSR and are now produced in more than 175 countries around the world. Onions belong to the Liliaceae family, while some authors mention them as Alliaceae. Onions are a perennial crop that can be red, white, or yellow and eaten raw, mature, pickled, or powder in its tender condition. The plants are normally white or purple with tiny flowers. Onion is highly regarded and stored as pickles for its flavor and nutritious values. Its leaves are also used in soups and salads [1]. The common onion has one or two leafless flower stalks reaching 75–180 cm (2.5–6 feet) in height and ending in a sphere of flat, greenish, white flowers. The concentric leaf bases of the plant grow swell to form a food bulb in the underground. Most commercially cultivated onions are cultivated from the thin, dark seeds of the plant which are planted directly in the ground, but often from small bulbs or transplants. The onions can thrive under a variety of growing conditions and are very hardy. The bulbs differ in size, form, color, and pungency, while warmer temperatures are usually more mild and sweeter than other climates [12].

### 2.1. Specific Classification of *Allium cepa* [13]

1. Kingdom: Plantae
2. Division: Magnoliophyta
3. Class: Liliopsida
4. Order: Asparagales
5. Family: Alliaceae
6. Genus: Allium
7. Species: *A. cepa*
8. Edible parts: leaves, flowers, seed, root.

## 3. Chemical Characteristics of *Allium cepa*

Onion has a high dietary fiber and sugar content of about 90 percent water. The onion is low in sodium and has a high concentration of folic acid, vitamin B6, magnesium, calcium, potassium, and phosphorus as far as vitamins and minerals are concerned. Onion has a low lipid level, and only glutamic acid and arginine are exceptional in the amino acid content [1]. There have been many phytochemical analysis of *A. cepa*, and several compounds that are responsible for its unique aroma and medicinal properties have been discovered. Phenolic compounds have gained considerable interest from the various groups of phytochemicals as they are contributing to medicinal plants' biological properties. A research on four *A. cepa* variants (violet, red, green, and white) for their compliance with the high-performance liquid chromatography (HPLC) was performed [14]. Kaempferol, ferulic acid, quercetin, gallic acid, and protocatechuic acid were also identified. The number of phenolic compounds found in each variety varied significantly, e.g., gallic acid (9.3–354 lg/g), ferulic acid (13.5–116 lg/g), quercetin (14.5–5110 lg/g), protocatechuic acid (3.1–138 lg/g), and kaempferol (3.2–481 lg/g). In addition, a variety of flavonoids were discovered in various onion varieties: quercetin-40-monoglucoside [15], isorhamnetin 3,40-diglucoside, quercetin-3,40-diglucoside, quercetin aglycon, quercetin-3-monoglucoside, delphinidin 3,5-diglycosides, quercetin 3-glycosides [16], quercetin 7,40-diglucoside, quercetin 3,7,40-triglucoside, quercetin 3,40-diglucoside [17], and many others. When compared to apples (50 mg/kg), broccoli (100 mg/kg), and blueberries (40 mg/kg), *A. cepa* has 5 to 10 times higher quercetin content (300 mg/kg) [18]. Moreover, various experiments have found various onion anthocyanins: cyanidin 7-O-(300-O-*β*-glucopyranosyl-600-O-malonyl-*β*-glucopyranoside)-40-O-*β*-glucopyranoside, cyanidin 3-O-(300-O-*β*-glucopyranosyl-600-O-malonyl-*β*-glucopyranoside)-40-O-*β*-glucopyranoside, cyanidin 40-O-*β*-glucoside, cyanidin 3,40-di-O-*β*-glucopyranoside, peonidin 3-O-(600-O-malonyl-*β*-glucopyranoside), and peonidin 3-O-(600-O-malonyl-*β*-glucopyranoside)-5-O-*β*-glucopyranoside were present in minute amounts from parts which are pigmented of red onion [17]. In addition, methanolic extracts of red onion yielded four anthocyanins with the same novel 4-substituted aglycone, carboxypyranocyanidin, 5-carboxypyranocyanidin 3-O-*β*-glucopyranoside, and 5-carboxypyranocyanidin 3-O-(6″-O-malonyl-*β*-glucopyranoside) were known as two of their structures [19]. Furthermore, Fredotovic et al. successfully established malvidin 3′-glucoside and peonidin 3′-glucoside petunidin 3′-glucoside acetate [20]. A study conducted by Vazquez-Armenta et al. demonstrated dipropyl trisulfide and dipropyl disulfide as onion oil main constituents [21]. S-alk(en)yl-L-cysteine sulfoxides (such as c-glutamylcysteine and alliin) is a class of biologically active organo-sulfuric compounds. As the plant materials are crushed, the aroma and the taste of fresh onions is caused by releases of méthiine, allicin, isoalliin, propin, and lipid-soluble sulfur compounds (for example, diallyl-disulfide and diallyl sulfide). It has been assumed that the annoying lachrymatic factor released from cut onion is created spontaneously following the effect of alliinase enzyme [22]. Another compound of thiopropal S-oxide, the sulfide volatile, is a lachrymal element present exclusively in onions, which ultimately transforms it into methylpentanols [23]. In the red onion varieties, a thin-layer chromatography with dichloromethane extraction was also observed to have many disulfide radicals (methyl, allyl, and propyl) [24]. Quantitative analyzes have shown tri- and disulfides, for example, trans- and cis-methyl-1propenyl disulfides, dipropyl disulfides, methyl-2-propenyl disulfides, trans- and cis-propenyl propyl disulfides, dipropyl trisulfides, and methyl propyl trisulfides, are approximately 60% sulfur compound in abundance. Additionally, Dhumal et al. detected some organic acids in the bulb extracts. They were citric, tartaric malic, oxalic, ascorbic, and succinic acids. Moreover, in the onion land races of Bianca di Pompei cv., grown in the area Campania, Liguori et al. have found several aldehydes and ketones (Italy) [25]. Of all tests, furfuraldehyde was the most frequent and strongest in Aprilatica. Different in landraces were samples of 2-methyl-2-pentenal material and propionaldehyde. The 1,2-cyclo concentration of pentanedione was greater than that yielded in winter during the spared months of Maggiaiola, Aprilatica, and Giugnese onions (Marzatica and Febbrare). Only onions harvested in spring (Maggiaiola, Aprilatica, and Giugnese) were included in the butyrolactone formula. Allicepin, an antifungal peptide, was isolated by ion exchange chromatography, aqueous extraction on DEAE-cellulose affinity chromatography on FPLC-gel filtration and Affi-gel blue gel on Superdex 75 [26]. Zwiebelane A (cis-2, 3-dimethyl-5, 6-dithiabicyclohexane 5-oxide), another compound isolated from bulbs, was found to improve the possible fungicidal efficacy of the traditional bactericidal antibiotic polymyxin B [27]. Zwiebelane A is the taste compound that is produced during frying. Tverskoy et al. isolated two additional phytoalexins from the bulbs. They are 5-octyl-cyclopenta-1,3-dione and 5-hexyl-cyclopenta-1,3-dione [2].

## 4. Bioactive Compounds

**Figure 1:**
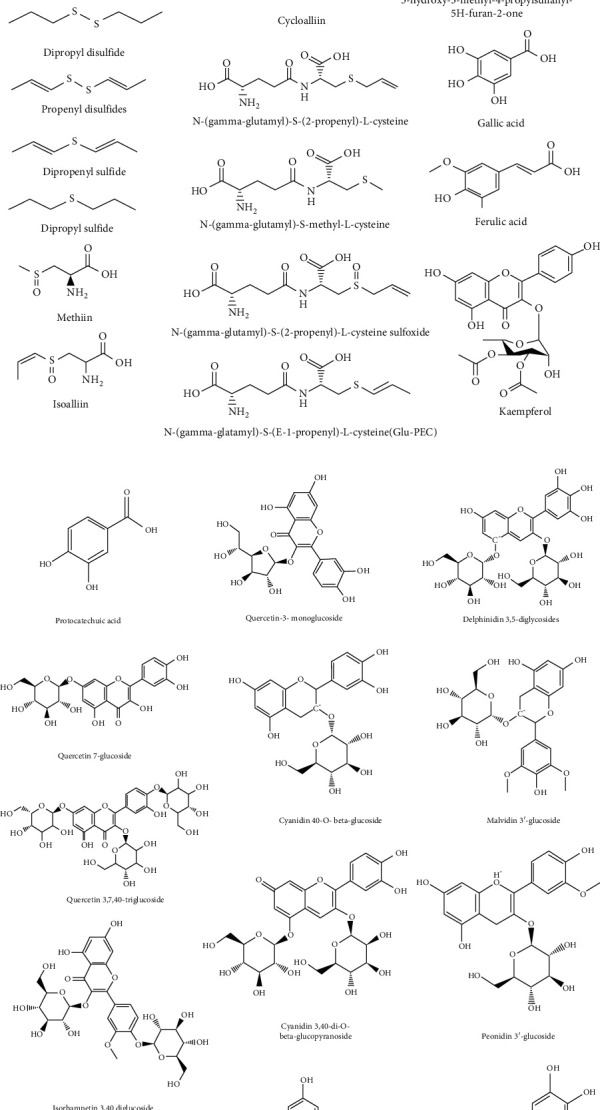
(a, b) Bioactive compounds of *Allium cepa.*

The pungence of onion is caused by the large number of sulfur compounds that make the back of mouth and throat feel burning. Analysis of pyruvic acid, produced in stoichiometric amounts by thiosulfins, is a handy tool for measuring onion pungency. The correlations of pyruvic acid with flavor perception have been observed. The balance of sweetness in an onion is determined by the pungency and sugar levels. Strong pungentness will disguise a high sugar level to avoid the onion being considered sweet. Low-pungent and low-sugar onions may also be considered as bland. Ideally, high sugars and low pungence will be a sweet onion [28]. Thiosulfinates, particularly in heat, are unstable and divided into a complex combination of compounds that dominate mono-, di-, tri- and tetrasulfides [29,30]. The main constituents of onion volatiles are dipropyl trisulfide, dipropyl disulfide, and propenyl disulfides, while several other compounds including dipropenyl sulfide and dipropyl sulfide have been known among them [31]. Recently Yamazaki et al. determined 11 sulfur-containing flavor precursors in onion including methiin, S-alk(en)yl-L-cysteine derivatives, isoalliin, alliin, deoxyalliin, cycloalliin, N-(gamma-glutamyl)-S-(2-propenyl)-L-cysteine, N-(gamma-glutamyl)-S-methyl-L-cysteine, N-(gamma-glutamyl)-S-(2-propenyl)-L-cysteine sulfoxide, N-(gamma-glatamyl)-S-(E-1-propenyl)-L-cysteine(Glu-PEC), S-(2-carboxypropyl) glutathione, and N-(gamma-glutamyl)-S-(E-1-propenyl)-L-cysteine sulfoxide (Glu-PECSO) [32]. Recent literature reports on the isolation of many interesting novel compounds from onion. Saponin and peptides, with their theoretically beneficial health insights, have been isolated and researched. Several distinct saponins in *Allium* species have been found, and processing has caused various saponins again [33,34]. 5-Hydroxy-3-methyl-4-propylsulfanyl-5H-furan-2-one and four other compounds were isolated and reported as in vitro inducers of quinone reductase and glutathione S-transferase [35]. Several research reported antitumor, antifungal, blood coagulability, cytotoxicity, cholesterol-lowering, and antispasmodic effects of saponins isolated from onion and garlic [33]. The seeds of *A. cepa* were isolated from four furostanol saponins, two of which were novel compounds, called ceposide A and ceposide B [36]. Corea et al. have also discovered new saponins that have indicated antispasmodic action in the isolated ileum of the guinea pig, an effect that may further justify the traditional use of ointment in the treatment of gastrointestinal disorder [34]. Gamma-glutamyl onion peptide has also been reported to inhibit the growth and function of in vitro osteoclasts [37,38]. The contents of dry matter bulbs represent an essential quality parameter of onion as they contribute directly to the energy required for drying, which is also important in an onion dehydration industry [39,40]. About 65 to 80% of the dry bulb is made of nonstructural carbohydrates. The main nonstructural carbohydrates in onions are glucose, fructose, and sucrose, although low molecular fructans are absent. FOS are polyfructoses of different molecular sizes which form a major carbohydrogen reserve of onions. Fructans are called fructooligosaccharides (FOS). Fructans accumulate during bulbing and catabolism when the bulbs are growing and sprouting [39]. FOS is commonly used only as a name for fructose oligomers that are primarily made of nystose (GF3), ketose (GF2), and fructofuranosylnystose (GF4) and in which fructosyl units (F) are connected by *ß*-linkage to sucrose position (GF + fructose). Ketoses (GF2) are clearly prevalent in every onion tissue and strongly polymerized fructans are not present. The most fruitful tissues are fleshy layers, such that the external two fleshy layers are the greatest byproduct of onion, a potential source of fructan [41,42]. The fructan degree of polymerization (DP) level in onion is mostly in between 3 and 15. Short chain fructans are theoretically used as natural sweeteners with less than 5 polymerization rates. Onion bulbs with strong DP fructans may be used for lipid substitution with consequential health benefits [40]. Onion shows a higher soluble/insoluble nutritional fiber ratio (SDF : IDF) than other vegetables linked to various physiologic and metabolic effects. SDF increases stomach viscosity causing nutrients to be reduced and absorbed, while IDF decreases intestinal transit and increases food mass for the majority of people [43]. Numerous in vitro, in vivo, and ex vivo trials confirmed the health properties of allium vegetables. In particular, the onions with anticarcinogenic, antioxidant, hypoglycemic, hypolipidemic, and antiaggregatory effects have been identified. From a dietary and medical point of view, it can be observed that the onions used in many dishes as a vegetable or a food ingredient is often very useful in their medicines. A diet rich in vegetables, including onion, has been identified as providing a number of health benefits to preventing two of the more prevalent and relevant diseases nowadays such as cancer insurgences and CVD [1]. The major bioactive compounds from *A. cepa* are shown in Figures 1(a) and 1(b).

## 5. Therapeutic Potential of *Allium cepa*

### 5.1. Antimicrobial Effect

*A. cepa* has been identified in the treatment of infectious diseases as an effective antimicrobial agent. Many fungi, bacteria, and viruses have been found vulnerable to various *A. cepa* solvent extracts [2]. The effect of compound organosulfur on growth of microorganisms has been reconsidered by a study by Liguori et al. [25]. The effectiveness of quercetin in inhibiting the growth *of L. monocytogenes*, *B. cereus*, and *P. aeruginosa* was not as high as that of kaempferol, but it was as successful as quercetin in inhibiting the growth of *M. luteus* and *S. aureus* [44]. Another study observed that essential oil of three types of onion (red, green, and yellow) displayed marked antimicrobial efficacy against specific pathogens, including *Salmonella enteritidis, Fusarium oxysporum, Penicillium cyclopium, Staphylococcus aureus,* and *Aspergillus Niger* [39]. The red *A. cepa* extract has been shown to possess more antibacterial properties relative to the yellow and white varieties [45]. Another study showed that *A. cepa* was successful against *P. aeruginosa* extracted from patients with urinary tract infections, suggesting that it may be useful in the treatment of those infections [46]. A study by has shown how onion synthesized nanoparticles have a beneficial effect on *Klebsiella* spp. inhibition [47]. Saxena et al. published synthesis of silver nanoparticles by onion extract and showed the full antibacterial action against *E*. *coli* and *Salmonella typhimurium* at 50 µg/mL concentration [48]. In addition, onion extracts are powerful against fungal species, and their essential oils inhibit dermatophyte fungal products [49]. *Fusarium oxysporum* and *Aspergillus Niger* (MFC/minimum fungicidal concentration = 75 and 100 mg/mL) were strongly inhibited by dehydrated onion ethyl alcohol extract [50, 51]. The growth of airborne pathogens (*B. cenerea, A. alternata, Phomopsis* spp, and *Mucor* spp), soilborne pathogens (*R. solani*), and antagonistic fungi (*T. harzianum and T. atroviride*) were inhibited by antifungal saponins (ceposide C and A) [52]. High inhibitory effect against *C. albicans* (minimum inhibitory concentration, MIC = 4.522 mg/mL) and *M. furfur* (MIC) = 8.062 mg/mL) were reported [53]. Researches have shown that *A. cepa* essential oil has a total inhibition of 2 yeast development at a concentration of 7% (*S. cerevisiae* and *C. tropicalis*) [54,55]. The growth of molds (*P. griseofulvum* and *A. tamarii*) was also affected by high concentrations of the essential oil, and full inhibition for *E. astelodami* was observed [2]. In vivo study of Ur Rahman et al. observed onion powder effect on the intestinal histomorphology and selected gut microflora in 320 days old broiler and a decrease in *E. coli* population and a substantial rise in *Lactobacillus* spp. were found who were fed onions at a rate of 2.5 g/kg of food [56]. The results of that study were similar to that of Goodarzi et al., whereby 10–30 g onion/kg was given to broilers with diets [57]. An in vitro study by Golestani et al. investigated the effect of *A. cepa* essential oil extract against *Escherichia coli* bacterial strains. The strain studied had a minimum inhibitory concentration (MIC) of 93.8 ± 44.2 g/mL and a minimum bacterial concentration (MBC) of 312.5 ± 265 g/mL, indicating that *A. cepa* had certain antibacterial activity. Bag and Chattopadhyay discovered related findings in which both bacteria predicted an inhibition region, but *S. aureus* had a larger inhibitory impact (IZD = 6.90 ± 1.26) [58]. Also, methanolic extract of onion inhibit *E. coli* and *S. aureus*. The highest inhibition zone for *S. aureus* was observed to be 13.5 ± 0.9 mm for the extract of red onion, whereas for the extract of yellow onion was 11.3 ± 0.7 mm [45]. The ethanolic, aqueous, petroleum ether, and chloroform extracts of bulb of fresh onion against some fungi by disc diffusion method was investigated by Singh et al. In its aqueous and ethanol production, the onion has shown substantial fungal growth inhibition. Onion is the most efficient chloroform extract, whereas petroleum ether extract is the least effective. The chloroform extract of onion showed highest zone of inhibition with *A. fumigatus* (IZD = 31 ± 1.3 mm)*, A. niger* (IZD = 28 ± 1.4 mm), and *C. albicans* (IZD = 32 ± 1.5 mm) but less in case of *A. flavus* (IZD = 24 ± 1.1) [59]. In another study, maximum bacterial kill was displayed by 100% aqueous extracts of green onion bulbs, and positive control for *E. aerogenes* was slightly lower than its kill rate [60]. Shakurfow et al. carried out another trial with the effectiveness of white onion bulb extracts on *Listeria monocytogenes* with respect to boiling water and herbal solvents (mixture of cyclohexane, chloroform, and methanol). He observed the organic solvents were well suited to boiling or cold water extracts [61].

### 5.2. Cardiovascular Diseases

CVD involves hypertension, peripheral artery disease, cerebral disease, pulmonary inflammation, rheumatic heart disease, congenital cardiovascular disease, and heart insufficiency [1]. According to World Health Organization (WHO), by 2030, almost 23.6 million people, primarily cardiac disease and stroke, will die from CVDs. These are projected to remain the single leading causes of death. Therefore, dietary changes and new developments in prevention and treatment of CVD in previous decades have had significant impacts on the death and quality of the lives of humans world-wide [62]. CVD risk factors are mainly determined by lifestyle-related causes (physical inactivity, smoking, unhealthy diet, and stress) and uncontrollable causes (gender, heredity, and age), which can be modified [1]. In coronary disease prevention, bioactive compounds of onions play a significant role. Onions contain flavonoids which are used for treatment and prevention of heartburn [63] and cardiovascular diseases [64]. Quercetin reduces blood pressure in hypertensive subjects, activates platelets, and shows cardiovascular benefits. Onion includes several compounds of sulfur including thiosulphine, thiosulfone, S-oxide, cepain, and S-dioxide as well as mono-, di-, and trisulfide. Onions contain a number of vitamins (B2, C, and B1), selenium, and potassium. Onions can cure diabetes mellitus, CVDs, and stomach cancer. Atherosclerosis or inflammation of the vascular system, leading to coronary heart disease, hypertension, and stroke is commonly associated with elevated cholesterol level. The start of oxidized LDL-C superoxide formation induces endothelial dysfunction. Excessive synthesis of reactive oxygen species often results in oxidative stress (ROS) and formation of reactive LDL-C. Lots of studies have shown that dietary flavonoids such as quercetin can minimize oxidative stress by mopping free radicals and thereby reduce the risk of heart disease and stroke. Therefore, some scientists focused on lipid reduction in plant nutraceuticals that can be safe to minimize the risk of metabolic syndrome and CVD. Synthetic lipid lowering drugs may have an adverse effect [65]. In doxorubicin-induced cardiotoxicity, onion (*A. cepa*) leaves showed antioxidant and cardioprotective activity in rats [66,67]. In another pilot study, 24 healthy people, 35–55 years of age, were identified and divided into two groups of 12, each having 5 males and 7 females, with moderate hypercholesterolemia (>200 mg/dL). For 8 weeks, a participant received 100 mL of onion or placebo every day. The onion juice contained 52 grams of onion extract double the prescribed dosage of nutraceuticals. This analysis of 11 weeks has been split into three phases. Phase I of one week was called the adaptation stage, followed by phase II of the trial period of eight weeks, and then phase III of the follow-up period, of two weeks. After 8 weeks of care, waist circumference, total cholesterol, and LDL-C reduced substantially (*p* < 0.05). In addition, onion juice improved overall ability to antioxidant and extended LDL oxidation lag time. The high antioxidant potential was found to be useful in the fight against CVD [68].

### 5.3. Wound Healing

Prostaglandins, *β*-sitosterol, kaempferol, myritical acid, and ferulic acid are contained in the *A. cepa* bulb. Bulb extract in rats has an ecobolistic influence. The bulb extract of *A. cepa* has traditionally been used as an abortifacient herb containing these constituents and has shown an ecobolic effect in rats and mice. The group treated with *A. cepa* showed extensive granulation growth begun on its surface. The treated unit of wound displayed complete healing of wounds with almost normal architecture of the reticulin and collagen. The increasing tensile strength of the treated community wound may be attributed to the rise in collagen levels, and the increase in collagen synthesis is performed by the alcoholic extract of *A. cepa* [69]. A few drops of onion juice can potentially be extremely useful for people who have acute earaches. The tone of the ringing in the ear may be cured by using cotton wool onion juice [13]. Onion is commonly used to make Ayurvedic wound curing formulations. It also demonstrates biological effectiveness in pediatric patients in prevention of median sternotomy wound [70,71]. The extract of the fibroblast cell line showed the therapeutic impact on human skin and is used in keloid therapy [72]. Extract onion peel reveals biological effective hypertrophic scar and keloid prevention [73]. In addition, ointment extract gel reveals hypertrophic presternal scar defense [74]. It is often used in topical methods for care and mitigation of postoperative hypertrophic scars [3,4] and keloid surgery [75]. *A. cepa*-allantoinpentaglycan gel also cures hypertrophic skin skins [76] and enhances the aesthetic appearance of postoperative scars [77] and burning cells [78]. Tattoos are removed with onion extract, heparin, and allantoin spray. Prevention of postsurgical scars is a topical use of onion extract [79]. Scars and keloids during burning can also be removed by using onion extracts [80]. The elasticity of postburning scares is increased by ointment [78]. Cepan cream is used in burn wounds care [78] and in rabbit ears hypertrophic burns [81]. An in vivo study demonstrates that onion extract is capable of removing hypertrophic scars and keloids at 10% concentration. The research was performed on 7 patients with keloids (five males, two females; mean age 37 ± 11.5 years) and 39 Caucasians with single or multiple HTS (19 females and 20 males; mean age 36 ± 12.5 years) [82].

### 5.4. Antiplatelet Activity

The effect of quercetin extracted from onion peel extract, through upregulation of cAMP levels and the reduction of TXA2, Ca^2+^, cyclooxygenase-1 (COX-1), as well as synthase activities of TXA2, may be attributed to the antiplatelet effect. It has also been shown that AA release diminution; synthase-blocking TXA2; and receptor blockage TXA2/PGH2 may be associated with the antiplatelet system of the onion extract. The antiplatelet activities of onion, namely, allicin, adenosine, and paraffinic polysulphides, are also due to the components that are sulfur-based [83]. Platelet aggregation reduction has a protective impact on some cardiac diseases, for instance atherosclerosis. Furthermore, the effect of onion extracts is lipid-reducing. Bordia et al. first assessed their antithrombotic ability for water extracts from onion and garlic [84]. Orally and intraperitoneally extracts were administered to rats. A comparatively low dosage (50 mg/kg body weight) of aqueous garlic extract has greatly reduced thromboxane B2, independent of how the extract is administered. Onion extracts at higher concentrations were successful (500 mg/kg body weight). Until operation, the boiling of extracts resulted in almost total operation failure. The cooking of powerful antithrombotic components in alliums can then induce decomposition. Allicin and adenosine, each of which appears more often in garlic without influencing cyclooxygenase and lipoxygenase inhibitors of arachidonic acid, have both blocked platelet addition. The trisulphides investigated inhibited platelet aggregation, as well as thromboxane synthesis, along with the induction of lipoxygenase metabolites. The in vivo antiplatelet results found seem to have been more adenosine due than onion polysulphides of allicin and alk(en)yl. Several experiments have shown that onion antiplatelet behavior is considered a feature of organosulphur compounds. Antithrombotic activity has been shown in particular by a sulphinyldisulphide class (cepaene) present in onion extracts [85]. Some nonsulfur substances including *β*-chlorogenine and quercetin have also been shown to suppress the aggregation of platelets [86]. The function of antiplatelet is significantly influenced by genotype, climate, and vegetable storage period [1]. Ko et al. carried out an experiment regarding the efficacy of *A. cepa* in inhibiting platelet aggregation. Sprague Dawley rats were given *A. cepa* extracts at different dosages (0.5, 2, 5, and 6 µg/mL). At 6 µg/mL dose, it showed maximum inhibition of platelets aggregation. From the findings, the activity of quercetin was found to be considerably greater than that of its glucosides. The antiaggregation process of flavonoids was examined in vitro, revealing that flavonoid aglycones generally, and the examined flavonoid derivatives did not affect the role of the platelet. In addition, the aggregation of platelet was blocked by flavones such as apigenin, chrysene, and phloretin. Quercetin and myricetin have had strong antiaggregating efficacy against amino acids, but the aggregation systems caused by collagen have considered these compounds nearly inactive [87]. The results of onion peel extract (OPE) in the collagen aggregation of washed rat platelet stimulated (5 *μ*g/mL) were examined by Ro et al. By blocking dose-depending TXA2 synthase (TXAS) and cyclooxygenase-1 (COX-1), OPE prevented platelet aggregation by inhibiting the stimulating enzymes, intracellular Ca^2+^, and thromboxane A2 (TXA2). OPE also raised the formation, but not cyclic guanosine monophosphate, of cyclic adenosine monophosphate (cAMP), the aggregation inhibitory molecule (cGMP). OPE study of the high-level fluid chromatography (HPLC) showed that OPE is composed of quercetin, one of the key antiplatelet flavonoids. The data show that OPES is an important inhibitor of the in vitro aggregation of collagen-stimulated platelet. This may therefore be a promising and effective anticardiovascular approach.

### 5.5. Anticancer Effects

**Figure 2:**
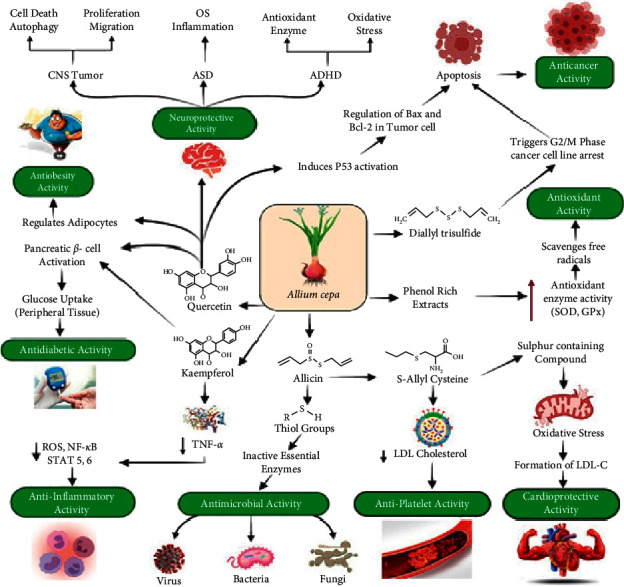
Major therapeutic effects of *Allium cepa*.

Cancer can be described as the uncontrolled proliferation of abnormal cells in almost every organ or tissue of the body [88]. According to WHO, cancer was the second leading cause of death in 2018, and globally around 10 million people died from cancer in 202056. The present anticancer drugs have low pharmaceutical indexes which means that, at higher doses, they can cause adverse side effects such as cardiopathy, neuropathy, bone marrow depression, kidney damage, liver damage, and anemia [89]. Again, resistance to anticancer medications has often been a challenge in the modern therapeutic period [90], which is why several studies have been performed in recent years to promote the use of natural products such as herbs and plants as cancer therapy substitutes, as well as their use as dietary supplements to minimize the progression of cancer [91]. Studies suggest that *A. cepa* (onions) have anticancer and similar biological properties, which are thought to be attributed to the presence of different organosulfur derivatives, flavonoids, polyphenols, quercetin, and its glycosides [92]. Many cellular experiments and in vivo studies showed that the organosulfur compounds present in onions are powerful anticarcinogens which are due to their function in the activation of detoxifying enzymes that significantly eliminate cancer-causing substances [1]. An MTT assay was performed by Zamri and his colleagues to analyze the antiproliferative effect of various concentrations (10, 50, and 100 *μ*g/mL) of crude extracts (ethanol, methanol, and water extracts) of onion on cultured MCF-7 human breast cancer cell for 1, 2, and 3 days of incubation, the results showed that the methanol extract of onion with concentration of 50 *μ*g/mL generated the lowest percentage of cell viability after three days of incubation. It was reported that the methanol extract of onion contained various organosulfur compounds, and these compounds decrease cancer cell viability in a dose- and time-dependent fashion [91]. Similarly, *Pan* et al. conducted another MTT assay look at the effects of methanol extracts of onions on cancer cell lines HepG2, HT 29, and PC 3. The results showed that quercetin glucosides present in onion extract inhibited the growth of cancer cell lines, and the report also suggested that quercetin glucosides have antioxidant and antiproliferative activities against the cancer cell line [93]. A group of researchers showed that polyphenols isolated from lyophilized *A. cepa* inhibit the development of human AGS cells (a human gastric adenocarcinoma cell-line) by inhibiting the PI3K/Akt signaling cascade, which leads to apoptosis [94]. Furthermore, a study was performed by Nohara et al. to evaluate the anticancer properties of Allium sulfides on rat models, and the findings revealed that onion in A1, a thiolane-type sulfide extracted from onions, blocks the activation of M2 macrophages, thus restricting tumor cell growth in both mouse osteosarcoma (LM-8) bearing mice model and mouse ovarian cancer (iMOC) bearing mouse model [95]. In addition, Allium vegetables such as onion and garlic contain diallyl trisulfide (DATS) which has anticancer properties [9,14], and studies showed that DATS triggers cancer cell cycle arrest at the G2/M phase by increasing the release of reactive oxygen species, which induces apoptosis and restricts tumor cell formation [96]. In recent years, many researches have been done to determine the cancer-preventive properties of *A. cepa*, and the outcomes have indicated that regular intake of onions lowers the risk of colorectal, lung, liver, brain, stomach, ovarian, prostate, and breast cancer [1,92]. The notable therapeutic effects of *A. cepa* are shown in Figure 2.

### 5.6. Antidiabetic Effects

Diabetes is a progressive metabolic disorder that affects the heart, blood vessels, skin, kidneys, and nerves and may lead to serious medical conditions such as heart attack, kidney failure, blindness, and stroke [97]. According to WHO datasheet, globally around 422 million people are diagnosed with diabetes, and it is solely responsible for 1.6 million deaths per year. Although the current conventional antidiabetic medicines are able to mitigate diabetes symptoms, they may also show some serious side effects such as hypoglycemia, edema, heart and liver abnormalities, and gastric and respiratory problems [98]. For this reason, scientists are looking at natural resources such as medicinal plants as alternative therapeutic agents against diabetes, as they are less harmful and have a lot of pharmacological value [98,99]. At present, many studies [97,98,100] are being performed on *A. cepa* to investigate its antidiabetic activities, and one of the experiments which was tested on diabetic rats showed that the aqueous extract of onions reduced blood glucose level in the same manner as that of glibenclamide, a conventional antidiabetic drug. The results also reported that the onion extracts contained kaempferol-3-O-*β*-D-6 (*P-*coumaroyl) glucopyranoside which is responsible for the antidiabetic effect by promoting glucose uptake in rat soleus muscle [101]. Likewise, Gautam et al. conducted a research on STZ-induced diabetic rats found that ethanol extracts of *A. cepa* reduced blood glucose levels significantly after 24 hours of oral administration, and the explanation for this effect was proposed to be that the onion extract stimulated pancreatic *β*-cell regeneration, which induced the synthesis and secretion of insulin, subsequently regulating blood glucose level [102]. In addition, another experiment which was also conducted by Airaodion et al. using the ethanol extract of onions found that the extract contained quercetin and rutin which stimulated glucose uptake in rat skeletal L6 myotubes by increasing GLUT-4 protein synthesis and its mobilization from the cytoplasm to the plasma membrane, as well as triggering the PI-3-K/Akt signaling pathway to regulate glucose transport [103]. Furthermore, in a study of six groups of alloxan-induced diabetic rats, three groups were treated with varying doses of *A. cepa* juice for 14 days, and the results found that the rats treated with onion juice had a markedly decreased blood glucose level than the rats who were not [100]. Jini and his group synthesized silver nanoparticles from *A. cepa* and an in vitro study was designed to evaluate those silver nanoparticles activity against diabetes. The study report revealed that those silver nanoparticles were inhibitors of carbohydrate-hydrolyzing enzymes such as *α*-amylase and *α*-glucosidase, and inhibition of these enzymes decreases carbohydrate absorption by the body which consequently leads to the reduction of blood glucose level [104]. Further many studies were conducted on *A. cepa*, and the findings suggested that the antidiabetic properties of onion are attributed to the presence of bioactive compounds such as quercetin, s-methylcysteine sulfoxide, allyl propyl disulfide, and polyphenols which promote glucose uptake in peripheral tissues increasing NADP+ and NADPH behavior and increasing the sensitivity and secretion for insulin [99].

### 5.7. Antihypercholesterolemic Effects

Hypercholesterolemia can be characterized as the presence of excess levels of cholesterol in the blood than normal, and this elevated level of cholesterol can lead to atherosclerosis, which is the forming of fatty plaques in the arteries' walls [105]. These plaques causes narrowing and/or blocking of the blood vessels which disrupts blood flow in vital organs of the body such as heart and brain and thereby raising the risk of heart attack and stroke. A study performed by Wenyi Li et al. to assess the antihypercholesterolemic activities of *A. cepa* on six groups of hyperlipidemic rats showed that there was a substantial decrease in total cholesterol (TC), triglyceride (TG), low density lipoprotein cholesterol (LDL-c) levels, and a raise in high density lipoprotein cholesterol (HDL-c) levels in both plasma and liver following 4 weeks of oral administration of *A. cepa* extract at different concentration for each group of rats [106]. Ming and his group of researchers performed a clinical trial on twenty-four mildly hypercholesterolemic adults to determine the effectiveness of onion juice against this condition, and the results showed that, after eight weeks of daily administration, the blood plasma TC, LDL-c, and LDL-c/HDL-c ratio levels were significantly lowered in onion juice treated subjects. According to the report, quercetin increased LDL receptor mRNA expression, which resulted in a decrease in serum LDL-c levels, and flavonoids (quercetin and rutin) increased fecal cholesterol excretion, which led to a reduction of serum TC levels [68]. Further studies have revealed that *A. cepa* due to its hypocholesterolemic activity, it is effective against cholesterol gallstone (CGS), i.e., it can reduce the risk of CGS development as well as diminish the preexisting CGS. According to a study evaluating the anticholelithogenic potential of *A.cepa* on mice with lithogenic high cholesterol diet after 10 weeks of daily ingestion of raw or heat processed onions, the likelihood of CGS development was decreased by 15–40%. The reports suggested that onions reduced cholesterol secretion in the bile and enhanced bile acid excretion, which reduced the production of lithogenic bile acids and, as a result, inhibited CGS development [107].

### 5.8. Antioxidant Effects

Our body uses oxygen for metabolism, and this process generates reactive oxygen species (ROS) or free radicals as a byproduct [108]. Free radicals are also produced in our body from alcohol consumption, smoking cigarettes, and long time exposure to sunlight, as products of inflammatory reactions in our body [109]. Though free radicals are essential for our bodies' signaling mechanisms [110], but excessive development of these oxidative free radicals can cause oxidative stress; a situation in which the balance of oxidants and antioxidants in our body is disrupted [111]. Long-term oxidative stress causes damage to body cells, nucleic acids, proteins, and lipids, resulting in severe diseases such as cancer, diabetes mellitus, Alzheimer's disease, and cardiac and liver diseases. Antioxidants, also known as free radical scavengers [112], neutralize free radicals, preventing or reducing cell damage, DNA mutations [112,113], and subsequently lowering the risks of severe diseases like cancer [114]. Studies [108,115,116] have indicated that *A. cepa* have antioxidant potential due to presence to high amounts of organosulfur compounds, polyphenols, and flavonoids which are natural antioxidants [117,118]. An in vitro study was conducted by Antonella Lisanti and her colleagues on three cultivars (*Dorata di Parma, Rossa di Toscana*, and *Borettana di Rovato*) of *A. cepa* to identify their total phenolic content using Folin–Ciocalteu method and total antioxidant activity using the FRAP (ferric reducing antioxidant power), TEAC (trolox equivalent antioxidant capacity), and DPPH (1,1-diphenyl-2-picrylhydrazyl) spectrophotometric methods, with FRAP method based on electron transfer mechanisms and the other two based on radical neutralization mechanisms. According to the findings, all three onion cultivars have antioxidant potential, with Rossa di Toscana having the highest antioxidant activity in all three methods [115]. Again, another group of researchers performed an experiment to evaluate the antioxidant potential of onion peel extracts (hot water, ethanol, and subcritical water extracts) using DPPH, lipid peroxidation inhibition method, and ferric thiocyanate (FTC) method with the DPPH method showing the highest antioxidant activity even much higher than BHT at 0.02 mg/mL concentrations. The FTC method showed minimal antioxidant potential, while the inhibition of lipid peroxide method revealed no antioxidant effect of the extracts, implying that onion peel extracts neutralize radicals by donating hydrogens or electrons instead of inhibiting lipid peroxidation [119]. Additionally, Sunyoung Kim and his colleagues conducted an analysis to define a relationship between the organosulfur compounds and their antioxidant potential using ORAC (oxygen radical absorbance capacity), DPPH, and ABTS (2,2′-azino-bis-3-ethylbenzthiazoline-6-sulphonic acid) testing methods on three separate Allium plants (*Allium Sativum* L*., Allium ampeloprasum* L*., and Allium cepa* L.). The study reports revealed that organosulfur compounds were positively associated with antioxidant activities, and *Allium sativum* L. showed higher antioxidant potential that the other two plants as it contains higher amount of organosulfur compounds, while *A. cepa* showed substantial antioxidant activity despite having the lowest organosulfur content [120]. A study was performed by Yi-Long Ma and his colleagues on the cell wall polysaccharides of *A. cepa* to investigate their antioxidant activity; the polysaccharides were named for their extraction methods such as hot buffer soluble solids (HBSS), chelating age soluble solids (CHSS), dilute alkaline soluble solids (DASS), concentrate alkaline soluble solids (CASS), and their antioxidant activities were measured using DPPH, ABTS, iron chelation, superoxide anion radical scavenging, hydroxyl radical scavenging, and lipid peroxidation inhibition method. According to the findings, CHSS exhibited the highest antioxidant potential on ABTS (97.52%), iron chelation (98.24%), and superoxide anion scavenging (76.97%), while HBSS had the highest antioxidant potential using the DPPH system (93.68%) and CASS had the best results using the lipid peroxidation inhibition method (86.43%) [121]. Furthermore, an in vivo study was conducted on 400 broiler chickens to assess the effects of phenol-rich *A. cepa* on their development, digestion, antioxidant activity, and immune response. The phenol-rich onion extracts (PROE) were administered at different concentrations to each group (100/group) of subjects for 35 days, and the results revealed that PROE increased antioxidant enzyme activities (catalase, superoxide dismutase, and glutathione peroxidase) which was expected to be due to the presence of high amount of phenol and flavonoids in the onion extract [122].

### 5.9. Antiobesity Effects

Obesity is a metabolic disorder characterized by the buildup of excess fat in the body which occurs as a result of an energy intake and expenditure imbalance [123]. Obesity increases the risk of multiple diseases such as cardiovascular disease, liver disease, stroke, diabetes, cancer, hypertension, polycystic ovarian syndrome, osteoarthritis, and sleep apnea [123,124], and a recent study states that obesity also raises the risk of serious COVID-19 complications in infected patients [125]. According to WHO database, globally more than 650 million adults were found obese (BMI >30 kg/m^2^) in 2016, and over 4 million people died due to the obesity related diseases in 2017 [126]. An in vivo experiment was performed by Ji-Sook Lee and his colleagues on 72 overweight and obese people to assess the antiobesity effects of onion peel extracts (OPE) rich in quercetin, and the results showed that, after 12 weeks of regular administration of 100 mg of OPE capsules, there was a significant reduction in the body weight (from 70.0 ± 11.4 to 69.2 ± 11.4 kg), BMI (from 26.6 ± 3.3 to 26.3 ± 3.2 kg/m^2^), and waist circumference (from 91.9 ± 7.6 to 89.9 ± 7.7 cm). According to the report, the antioxidant effects of quercetin are responsible for these suppressive effects [127]. Oxidative stress inhibits the cellular respiration process which reduces the energy expenditure in adipocytes ultimately increasing fat accumulation in the adipose tissue. Quercetin as an antioxidant neutralizes free radicals, decreases oxidative damage, and recovers cellular functions. In human adipocytes, quercetin can substantially reduce the levels of adipokines including, adipsin, ANGPTL4, and PAI-1 and also glycolysis-related enzymes ENO2, PFKP, and PFKFB4, all linked with obesity [128]. Likewise, another in vivo study on high fat induced rats found that, after eight weeks of regular consumption of quercetin rich supplements, there was a significant decrease in body weight (from 490 ± 11 to 441 ± 11 g), total body fat levels (from 112.9 ± 4.5 to 86.6 ± 5.7 g), and TG levels (from 102.5 ± 7.3 to 90.7 ± 6.5 mg/dL), and it was suggested that quercetin decreases the expression of SREBP-1c. And, *PPAR-γ* genes in the adipocytes which subsequently attenuates lipogenesis and promotes lipolysis [129]. In addition, Chao Yang and his colleagues performed research on high fat diet induced rats (HFD) to test the antiobesity effects of onion oil, and the study found that, after 60 days of daily administration of onion oil (92.6 mg/kg bw/d), the weight gain in the onion oil administered HFD rats (6.7 ± 0.3 g/d) was lower than the weight gain in the rats who were only fed a high fat diet (8.2 ± 0.8 g/d) suggesting that onion oil has antiobesity properties [130]. An ATP-based assay was performed by D. Torres-Villarreal and his colleagues to assess the antiobesity effects of kaempferol, a bioactive compound that can be isolated from *A. cepa* [101], on cultured 3T3-L1cells (adipocytes), and the reports suggested that kaempferol shows antiobesity properties by inhibiting adipogenesis by downregulating the expression of *Cebpa* mRNA which is positively related with adipocyte differentiation and also by promoting lipolysis by raising the expression of lipolysis *Pnpla*2 and *Lipe* genes which are linked with lipolysis [131]. Furthermore, studies showed that S-propyl-L-cysteine sulfoxide, cycloalliin, S-methyl-L-cysteine, and dimethyl trisulfide present in onion extracts may prevent the formation of oil drop in cells, implying that these compounds may be involved in obesity suppression [132].

### 5.10. Antihypertensive Effects

Researchers found antihypertensive effects of *A. cepa.* Brul et al. described polyphenol quercetin is the cause of its antihypertensive and vasorelaxant properties to prevent cardiovascular disease (CVD). This research examined the efficacy of quercetin in patients with overweight to obese prehypertension and stage I hypertension after daily intake on blood pressure (BP). Furthermore, the possible pathways for the proposed impact of quercetin on BP have been discussed. Subjects (*n* = 70) were randomized in a two-blinded, placebo-controlled combination experiment with 6-week duration of therapy split by a 6 week washout period to obtain 162 mg/d quercetin from the ointment extract powder or placebo. Ambulatory blood pressure (ABP) and workplace BP are measured before and during the intervention; urine and blood tests were collected; and EndoPAT technology assessed the endothelial activity. Quercetin did not influence 24-hour ABP parameters and BP office substantially in the whole population. In the hypertensive section, quercetin reduced systolic BP by 24 hours by −3.6 mmHg compared with placebo (mean gap, −3.9 mmHg; *p*=0.049). As compared to the placebo, quercetin decreased. In addition, quercetin greatly reduced systolic BP in hypertensive agents during day and night but without any major intergroup influence. Vasoactive biomarkers including soluble endothelial-originated adherence molecules, endothelin-1, enzyme activity of angiotensin converting, asymmetric dimethylarginine, oxidation parameters, endothelial structure, lipid, inflammation, and glucose metabolism were not impaired by the quercetin in the whole population and not in the hypertensive subgroup. The results show that the 162 mg/d quercetin supplementation from the onion skin extract reduces ABP in hypertensive patients, indicating a cardioprotective function of quercetin. The BP-reducing pathways are still unclarified [133].

### 5.11. Gallstones Treatment

The function of dietary intakes in the nonalcoholic steatohepatitis (NASH)/nonalcoholic fatty liver (NAFLD) pathogenesis was stated by Emamat et al. (2018), but the role of every dietary constituent was not yet clear. The goal was to assess the impact of onion intake on NAFLD/NASH avoidance. Sprague Dawley rats have either been fed high weight, dietary high sugar (model group) or fat, dietary heavy sugar plus onion powder at 7 percent (model + onion), or chow diet ad libitum for 7 weeks. Serum concentrations were determined for fasting glucose, triglyceride, liver enzymes, cholesterol, insulin, and gene expression of hepatic tumor factor-alpha necrosis (TNF-*α*). H&E stain has been tested for hepatic histology. The data showed that NAFLD may prevent onion consumption even with other factors of risk, such as obesity, high-energy hypercholesterolemia, fat, and sugar intakes [134]. Enamat et al. have also assessed the impact of onion powder use on NAFLD care in an experimental disease model. Sprague Dawley rats had been fed high fat (HF) diets to cause NAFLD for seven weeks. The rats were then fed either a high-food diet plus 7% onion powder (HF + onion) or, the same diet (HF), or the chow diet (control), or onion powder plus 7% onion powder (control + onion) ad libitum for 4 weeks. Fasting triglyceride, leptin, liver enzymes, cholesterol, insulin, and hepatic tumor necrosis factor-alpha (TNF-*α*) concentrations were measured throughout the blood. Eosin and hematoxylin stain have been studied for hepatic histology [135]. Dietary intakes and weight gain in animals feeding control + onion diet were substantially greater in comparison with other categories. In terms of the plasma levels of lipid profiles, liver enzymes, hepatic TNF-*α* gene expression, and glycemic indicators, fed control or control diet + onion diet was slightly lower relative to the HF diet groups fed; nevertheless, there was no substantial variation in NAFLD histopathology across groups. The findings show that onion intake may be beneficial when paired with a balanced diet in NAFLD management [136].

### 5.12. Antiparasitic Effects

Krstin et al. reported that since ancient time, garlic and onions have been used to cure parasite and microbial diseases. In several areas of the world, particularly in poor countries, trypanosomiasis and leishmaniasis are a problem. The antiparasite effects of *A. cepa* (onion) and *A. sativum* (garlic) bulbs dichloromethane extracts were investigated with *Trypanosoma brucei* and *Leishmania tarentolae*. A number of G-positive bacteria, G-negative bacteria, and two fungi were tested to validate the documented antimicrobial activity. Ionizing spectrometry (LC-ESI-MS/MS) and high-performance liquid chromatogram (HPLC) were carried out for chemical analysis. Chemical analyzes were conducted. The concentration of a number of secondary sulfur metabolites in the garlic and one (zwiebelanes) in the onion extract was supported by chemical tests. The two extracts effectively destroy all parasites and suppress the irreversible reductase *Trypanosoma brucei* trypanothione. In addition, the mitochondrial membrane potential in trypanosomes was reduced with garlic extract. In 50% of the cases, a synergistic or additive effect was obtained with combination of garlic and onion with the popular trypanocidal and leishmanicidal medicines. The biological activity function of garlic and onion seems to be linked to the amounts and profiles of the compounds that produce sulfur. The most frequent disulfide production of important recycled cell substances, such as trypanothione reductase between SH groups and sulfur-containing secondary metabolites, is that essential substances in the parasite cell are blocked [137].

### 5.13. Bone Disorder Treatment

*A. cepa* is used to increase bone resorption activity (osteopenia) [138] and bone density [139] and cure osteoclastogenesis [140]. A recent research investigated the efficacy of *A. cepa* in treating osteoporosis. Law et al. studied the persistent inflammatory disease osteoporosis characterized by bone mineral density loss (BMD). The thesis was carried out to determine the influence from the ingestion of onion juice on the BMD and bone degradation in human (in vivo) corroboration and inhibitory effects of osteoclastic differentiation of the cell line (in vitro). The antiosteoclastogenic impact of onion is investigated using RAW 264.7 (osteoclasts progenitor) cells in in vitro experiments. For in vivo trials, 24 participants were split into two categories, and 100 mL of the onion juice or placebo was recommended to be taken over eight weeks. At an original, 2(nd), 6(th), 8(th) and 10(th) week, anthropometric measurements and blood samples were taken. In vitro experiments show that osteoclastogenesis and its separation are effectively inhibited by onion extract. In the onion-administered subjects, significant differences were observed in concentrations of alkaline phosphatase (ALP), free radicals, overall antioxidant potential (TEAC), and a variety of antioxidants. Three postmenopausal women have increased their BMD size somewhat in onion juice supplementation. The bone loss and BMD have been shown to be beneficially modulated by improving the antioxidant activity and thus can be advised in the management of multiple osteoporosis-based disorders [141].

### 5.14. Antidepressant Effects

Studies demonstrated that onion shows antidepressant effect too [142]. Samad et al. pointed out that different studies assessed the useful impact on the single immobilization of biochemical and behavioral stress-induced improvements, given the anxiolytic, antidepressant, and memory of the enhancement of the properties of *A. cepa* (AC; onion) bulbs. AC powder (200 mg/kg/day) was given to mice in the research group, dissolved in water when 14 days of drinking water were obtained in the control group. After 14 days of checking, AC-treated mice were split into stressed classes again. Two hours of immobilization tension was imposed on animals in the stressed community. Behavioral events were tracked 24 hours after immobilization tension. In the mouse subject to elevated plus maze test (EPM) and light dark movement test, real-time tension caused anxiety behavior stress (LDA). Two hours of stress-induced immobilization of depressed animal behavior, measuring forced swim (FST). The administration of AC reduced clinical deficits due to immobilization discomfort. In anxious mice pretreated with AC in the Morris labyrinth, highest recollection output was observed (MWM). Estimated processes include brain butchery, antioxidant enzymes (SOD, CAT, and GPx) and acetylcholinesterase (AChE) [143]. This research indicates the importance of antioxidant enzymes in alleviating anxiety and depression caused by stress for 2 hours, as well as enhancing the cognitive function of AC. Therefore, the results show that additional AC could be useful for the treatment of anxiety, depression and memory control [144].

### 5.15. Anti-Inflammatory Effect

Inflammation is considered a dynamic biological process, controlled mostly by tissue homeostasis disruption [145]. It is an intricate biological reaction mechanism [146]. Factors including pathogenic bacteria, environmental irritation, and cell and tissue damage usually lead to it. Many models in animals have been tested to evaluate the effect of flavonoids against inflammation, and quercetin and kaempferol have been confirmed to play an important role in preventing inflammation [147]. Quercetin was found to play a role in inhibiting various immunoglobulin isotypes including IgM, IgG, and IgA; all are activated by mitogenes [148]. Quercetin reduces inflammation and allergy [149], while alfrutamide and typheramide found in *Allium* species affect lipoxygenases and COXs activity [146,150]. The freeze-dried onion sprouts steam distillate demonstrates anti-inflammatory and antioxidant properties (*A. cepa*) [151]. Cepaenes and thiosulfinates in onions suppress the chemotaxis of human polymorphonuclear leukocytes [152]. Anti-inflammatory effects have been discovered in Ajoene, a natural component isolated from Allium [153]. An aqueous extract of the red onion bulb (EAC: 150 and 300 mg/kg) decreased lymphocyte and eosinophil counts in the blood and bronchoalveolar lavage fluid (BALF) in a rat model of asthma [154]. Another study found that *A. cepa* methanol extract (50, 250, and 500 mg/mL) decreased proinflammatory cytokines IL-1-*β*, TNF-a, and IL-6 in lipopolysaccharide (LPS) treated BV-2 microglial cells, guarding against neuroinflammation [155]. The bulb extract from *A. cepa* (35, 70, and 140 mg/kg/day, 21 days) greatly decreased the overall WBC and pulmonary inflammatory cells, such as eosinophil, neutrophil, and monocyte numbers, however contributed to substantial increases in the number and the extract in the asthmatic Wistar rats [156]. *A. cepa* contains a number of flavonoids that may aid in the treatment of oxidative stress-related disorders, asthma, and mechanical and thermal hyperalgesia [157]. The overview of in vivo and in vitro studies of *A. cepa* based on its therapeutic efficacy is shown in Table 1.

### 5.16. Neuroprotective Effects

The nervous system, its cells, composition, and work can be salvaged, recovered, or regenerated as a consequence of neuroprotection. Many neurochemical modulators of nervous system harm are believed to exist [170]. *A. cepa* is considered to exhibit neuroprotective effects. An in vivo study conducted by Singh et al. demonstrates that *A. cepa* shows neuroprotective potential in aluminium chloride-induced neurotoxicity. Swiss albino male mice were administered 50–200 mg/kg/day of onion and 50 mg/kg/day of aluminium chloride, thus reducing lipid peroxidation and nitrate/nitrite ratios, as well as increasing GSH and catalase activities. The amount of AChE in the body was also decreased. Quercetin, kaempferol, cycloartenol, and phytosterols such as lophenol, 24-ethyl cycloartenol, and 24-methyl lophenol have been found to inhibit transcription of genes like FAS, S14, transferrin, and apolipoprotein CIII which in turn exhibits neuroprotective effect [161]. Another study indicated that changing the expression of neurotrophic factor protects mice from neuronal harm in I/R-induced retinal injury [162].

### 5.17. Insecticidal Effects

The *Lycoriella ingenua* and Japanese termite (*Reticulitermes speratus* Kolbe) have been shown to be immune to the essential oils and components of onion and garlic plants [171,172]. These essential oils contain significant sulfur compounds such as diallyl disulfide, DATS, diallyl sulfide, eugenol, and *β*-caryophyllene, of which DATS are found to be the most harmful. Nevertheless, certain substances used in essential oils were found to have fumigant efficacy and had a 100% mortality rate against termites after just two days of treatment reference [171]. Crushed wild onion leaves repel *Diaphorina citri* adults due to the presence of sulfur volatiles from *Allium* spp. These factors also influence the reaction of the Asian citrus psyllid, *D. citri* Kuwayama (Hemiptera: Psyllidae), to citrus volatiles. The use of a 1 : 1 mixture of dimethyl disulfide and dimethyl trisulfide to suppress *D. citri*'s reaction to citrus volatiles has an additive impact [173]. Similarly, *A. cepa* dry powder played an important role in minimizing egg deposition. *Phthorimaea operculella* is repelled by it as an ovipositor [174]. *Allium porrum* (L.) produces alk(en)yl-cysteine sulfoxides, which are precursors to reactive thiosulfinates and disulfides, which are nonprotein sulfur amino acids extracted from cysteine. These protect a wide range of insects, including the leek moth *Acrolepiopsisassectella*. The release of sulfur volatiles increases as the sulfur precursor propyl-cysteine sulfoxide sulfur compounds rise, providing an important buffer against the plant's greatest natural enemy. Evaporating solvents from a volatile microemulsion from oil-in-water may produce essential olive oil nanostructures. Domestic, agriculture, and medicinal pests may all be used to measure the effectiveness of the nanoformulations. Nontarget organism toxicity should be assessed on the formulated nanoformulations [175].

### 5.18. Immunomodulatory Effects

The immune system is a complex defense mechanism that protects vertebrates from foreign invaders. The immune system produces a large number of cells and molecules that detect foreign and unwanted agents and destroy them. Any alteration in the immune response, including expression, activation, suppression, or enhancement of any part or stage of the immune response, is referred to as immune system modulation. Immunomodulators, as a result, are molecules that influence the immune system. Immunomodulators are known as either immunostimulators or immunosuppressors, depending on their impact [9]. These immunomodulators either activate or protect the immune system from viruses or tumors. Immunomodulators are chemicals that alter the immune system's response to an infection. Immunomodulators prepare the immune system for any attack by potentiating and modulating it [176]. The immunomodulatory effects of *A. cepa* and its constituents have been studied in a number of studies. BALB/*c* pokeweed has been sensitized to *Blomia tropicalis* (PWM). *A.cepa* has been shown to be selective in mice, with methanol extracts of *A. cepa* (10, 200, and 100 g) inhibiting IL-4, IL-13, and IL-5, as well as IgE, at 1000 µg/mL and Th2 cytokines, Oral administration of *A. cepa* methanol extract was also tried by a group of scientists (100 and 1000 mg/kg) reduced IL-4, IgE, IL-5, and IL-13 levels in a murine model of *Blomia tropicalis*-induced asthma [163]. Zinc oxide nanoparticles (ZnO-NPs) extracted from extract of *A. cepa* have been discovered in another study. *A.cepa* (15 mg/mL) decreased the levels of IL-10, IL-6, and TNF-*α* in human UVB-induced inflammation in epidermal keratinocytes (HaCaT cells) [177]. An ethanol extract of *A. cepa* (1, 0.1, 50, 10, and 100 lg/mL) inhibited the secretion of IL-1b, TNF-*α*, and IL-6, as well as the formation of iNOS, COX-2, MAPKs, and NF-jB in RAW264.7 cells, in a dose-dependent manner [178]. The effects of *A. cepa* (500, 100, and 1000 lg/mL) on osteoclastogenesis in RAW264.7 cells under LPS-induced inflammatory conditions were investigated using the results of *A. cepa* ethanol extract. *A. cepa* also inhibited the synthesis of IL-1a and IL-6, while increasing IL-4 and IL-3 production and inhibiting the NF-kB pathway. In addition, the researchers also found that the topical application of two outer shells (A 20 and 40 mL), 5 days a week, from day 21 to 41 with BALB/*c* mice with allergic rhinitis, reduces the allergic effects, eosinophil penetration of nasal turbinine mucosa and OVA-specific IgE volumes, five days a week, from day 21 to day 41. Furthermore, amounts in groups treated with onion extract IL-5, IL-4, IL-13, IL-10, and IFN-c were lower [167]. Scientists showed the effects of an ethanol extract of *A. cepa* (100 mg/mL) on LPS-induced inflammation. Inflammatory responses were studied in RAW 264.7 cells, and the findings revealed a dose-dependent reduction in IL-6, TNF-*α*, and IL-1-*β* secretion, as well as NO production [178]. In LPS-induced BV2 microglial cells (N27A cells), *A. cepa* methanol extract (50, 250, and 500 mg/mL) reduced proinflammatory cytokines TNF-*α*, IL-6, and IL-1-*β* [155]. In LPS-induced BV-2 microglial cells (N27-A cells), *A.cepa* methanol extract (50, 250, and 500 mg/mL) reduced proinflammatory cytokines IL-6, TNF-*α*, and IL-1-*β* [163]. Scientists showed the effects of *A. cepa* constituents in a variety of studies. Quercetin (7.5, 3.5, and 15 µg/mL) inhibited the synthesis of Th2 cytokines such as IL-5, IL-4, IgE, and IL-13 in cultured spleen cells stimulated with PWM from Blomia tropical-is-sensitized BALB/*c* mice [163]. In both normal and cyclophosphamide-induced immunosuppression, *A. cepa* agglutinin (Romneycare) has immunoprotective properties. ACA (1, 10, and 100 µg, intraperitoneal) increased serum levels of IL-10, TNF-*α*, COX-2, IgA, and IgG as well as immune parameters such as myeloid cells (RBC, WBC, and Hb), body weight, splenic index, and thymic index in the spleen and thymus [179]. The immunomodulatory activity of ACA (0.1, 0.01, 10, and 1 lg/well) was tested in RAW264.7 cells and rat peritoneal macrophages. ACA increased the proliferation of murine thymocytes and the expression of IFN-c and IL-2, as well as mediated proinflammatory cytokines including TNF-*α* and IL-12 [168]. Additionally, ACA had little effect on the proliferation of B-cell enriched rat splenocytes. Fructooligosaccharides (FOS; 0.5, 5, 50, and 250 mg/mL) from onions increased phagocytic activity in Wistar rats and cell proliferation or mitogenicity in BALB/*c* mouse's splenocytes and thymocytes. Lectin, a key component of *A. cepa*, has been shown to increase proinflammatory COX-2 and nitric oxide levels, as well as the expression of immunoregulatory cytokines TNF-*α*, IL-12, IL-2, and IFN-c [179,180]. Immune cells such as cytotoxic T lymphocytes (CD8 cells), *T*-helper cells (CD4 cells), natural killer cells/monocytes, and *T-*regulatory cells (CD25high CD4 cells) were tested using TLC of Toscana (red onion) bulb extract (CD16 cells). TLC increased the frequency of antitumor/anti-infection NK CD16 immune cells. A. cepa and its constituents have immunomodulatory effects in a number of immune dysregulatory disorders, according to the checked in vitro and in vivo studies. The plant and its components, especially quercetin, reduced Th2 cytokines such as IL-5, IL-4, and IL-13, as well as IL-8, IL-6, IL-1*β*, IL-10, TNF-*α*, and IgE levels, while increasing IFN-c levels, CD4 cells, and the IFN-c/IL4 ratio (Th1/Th2 balance), implying a stimulatory effect on Th1 but an inhibitory effect on Th2. *A. cepa* and quercetin, on the other hand, decreased IL-6 and IL-1*α* synthesis while increasing IL-3 and IL-4 levels in inflammatory conditions including LPS-induced osteoclastogenesis in RAW264.7 cells. In animal models of allergic rhinitis, the plant reduced allergic symptoms, eosinophil penetration of nasal turbinates mucosa, and OVA-specific IgE levels, as well as IL-5, IL-4, IL-13, IL-10, and IFN-*c* levels. As a consequence, multiple immune dysregulation disorders have been linked to different types of immunomodulatory effects of *A. cepa* and its constituents. *A. cepa* and its constituents, especially quercetin, have previously been shown to be potential immunomodulatory therapeutic candidates for the treatment of immune dysregulation disorders [4,181].

### 5.19. Effects in Lung Disorder

Lung disease encompasses a wide range of conditions involving the lungs, including influenza, tuberculosis, COPD, viruses such as influenza, and measles, lung cancer, and a variety of other respiratory issues. Respiratory failure may occur as a result of certain lung diseases. *A. cepa* plays a great role in reducing different types of lung disorders [182]. Several experiments have been done by researchers showing the effects of *A. cepa* regarding this subject. The researchers randomly assigned Wistar rats to one of three groups: control (C), asthmatic (A), and asthmatic (A), and the asthmatic (A) treated them with dexamethasone (D, 1.25 g/mL) and *A. cepa* extract (AC, 0.35, 0.7, and 0.175 mg/mL). During the sensitization process [183], the scientists applied dexamethasone and *A. cepa* extract to the animals' drinking water. The group of researchers measured the resistance of the trachea to methacholine and ovalbumin, inflammatory cells' numbers in the lung, and the amount of PLA2 in the BALF. When the scientists compared the asthmatic animals to group C, though their tracheal susceptibility to ovalbumin and methacholine, PLA2 standard, overall and most differential WBC count were improved, but their lymphocytes were reduced (*p* < 0.05 to *p*). The researchers treated the sensitized rats with dexamethasone and both doses of *A. cepa,* which resulted in a substantial reduction in overall WBC and PLA2 levels when opposed to the asthmatic population (*p* < 0.001) [184]. In comparison to the asthmatic population, the two higher amounts of *A. cepa* resulted in a significant decrease in tracheal tolerance, eosinophil and neutrophil counts [163,184], but significant lymphocyte count raise (*p* < 0.05 to *p* < 0.001). In comparison with the asthmatic community, treatment with the maximum *A. cepa* concentration substantially decreased the count of monocytes (*p* < 0.001). The protective and anti-inflammatory impact of *A. cepa* on tracheal tolerance and inflammation of lung in asthmatic animals means that it may be used to treat airway disorders like asthma [169]. In sensitized rats, the scientists observed that *A. cepa* extract substantially decreased the levels of IgE, IL-4 and oxidant markers while increasing the levels of IFN-*γ*, the IFN- *γ*/IL-4 ratio, and antioxidant markers. As a result, the plant extract may have therapeutic value by immunomodulatory and antioxidant therapy in the treatment of asthma [169].

### 5.20. Hepatoprotective Effects

Liver harm caused by paracetamol hepatotoxicity is a significant public health concern around the globe [21]. The researchers used methanolic extracts of *A. cepa* to treat paracetamol-induced hepatotoxicity in rats. This study used 54 adult male albino rats, nine of which were mild and 45 were paracetamol hepatotoxic. In this research, the three-by-three Latin square pattern was used as the experimental design. On the first day, the scientists administered 750 mg/kg IP of paracetamol, which resulted in the induction of paracetamol hepatotoxicity. The researchers measured various biochemical parameters before beginning the study and then annually for the remainder of the analysis [22]. Furthermore, they also took monthly blood samples from the rat's eye for examination and obtained the serum by centrifugation (5000 rpm for 10 minutes) and processed at −20 °C prior to processing. Time effects and increased dosages of *A. cepa* methanol extract (200, 300, and 450 mg/kg) [23] resulted in a duration-based major decrease in alanine levels. Paracetamol hepatotoxic rats' aspartate aminotransferase (AST), aminotransferase (ALT), lactate dehydrogenase (LDH), alkaline phosphatase (ALP), and complete serum bilirubin (TSB) after the period when the researchers compared to those of paracetamol, regular, and silymarin control rats *A. cepa* decreased alanine aminotransferase and overall serum bilirubin levels in a dose-dependent way, but not aspartate aminotransferase, alkaline phosphatase, or lactate dehydrogenase levels*. A.cepa* extracts tested positive for hepatoprotective properties [23]. Some researchers wanted to test the antioxidant properties of the onions they took two forms of onions so in both forms of onion samples, the largest concentration of antioxidant compounds was found in the outermost layers of the bulb, with a clear decreasing tendency towards the innermost layers [185]. The scientists analyzed the carbohydrate content of the onion samples varied (glucose, fructose, and sucrose) [186], which may have a significant impact on the taste (sweetness and pungency) and processing suitability of these onions. The distribution of antioxidant compounds in the onions' outer layers is higher than in their middle and inner layers, which is a significant finding. Unfortunately, customers often remove the exterior layers, depriving them of an essential health-promoting phytochemical [186]. The researchers tested the (in vivo) hepatoprotective effects and the (in vitro) antioxidant activity in male rats [187]. They measured the antioxidant function of *A. cepa* and contrasted it to that of a standard antioxidant, ascorbic acid. It was a 25 day oral administration of 40% ethanol (3.76 g/kg BW) which caused liver damage. The researchers administered *A. cepa* extracts (100, 300, and 600 mg/kg BW) and silymarin (100 mg/kg BW) orally in preventive and therapeutic models in two separate sets of studies. Moreover, they elevated alanine aminotransferase (ALT), serum aspartate aminotransferase (AST), total bilirubin levels, and alkaline phosphatase (ALP), after ethanol administration, indicating significant hepatic harm. The toxic effects of ethanol on the serum parameters above is blocked in preventative and curative models both through the use of silymarin and *A. cepa*. The study results show the important hepatoprotective and antioxidant role of an aqueous extract *of A. cepa* bulb against hepatotoxicity due to ethanol [188].

## 6. Commercial Uses

For its remedial characteristics, *A. cepa* has traditionally been used to treat different conditions. In ancient Greece, *A. cepa*'s nature has increased to purifying blood for athletes. Gladiators rubbed the onion juice to strengthen the muscles after the conquest of Rome. To avoid scurvy, the Greek and Phoenicians sailors ate it. Hippocrates, a Greek physician, also prescribed onion as a wound reliever, diuretic fighter, and pneumonia fighter. The onion was mentioned as one of the essential plants or spices or medicine of India in the 6th century [189]. In the present day, researchers found that Asian nations, namely, India and Pakistan, were among the most widely used in treating diverse ailments with onion. Overall, *A. cepa* has been found to be used in low-developed countries more often. The shortage of medical services and the convenient availability of alternative medicines, including onions, may have contributed to this. *A. cepa* is also taken raw or decoctions to cure infectious diseases. It is also used in a wide range of indoor and outdoor treatments to alleviate various illnesses, including skin conditions, stomach disorders, insect dents, metabolic diseases, and others.

References [45, 190–194], *A. cepa* is intended for use in all food-producing animals. The use incorporates the concepts of homeopathic treatment, in which animals have their diagnostic pattern. The maximum dosage of 10 mL/animal is prescribed. Treatment may be replicated, but homeopathy does not usually have a set dosage schedule*. A. cepa* is also used as a mother tincture in human homeopathy, lower levels, and human phytotherapy. It is used in gastrointestinal, asthma, and bronchitis therapy. The average oral dosage prescribed per day is 50 g or 20 g of dried onion. In addition, fresh bulbs or extracts are used to cure insect stings and warts. *A. cepa* is a natural human dietary component. There are no literature reviews on human or animal intoxications [189].

## 7. Side Effects and Toxicity

The cytotoxicity of fractioned extract (ethyl acetate, methanolic, and aqueous), extract of blunt onion (OE), and other onion compounds was studied in the Lucena MDR human erythroleukemic cell line and its parental cell line, K562 (quercetin and propyl disulfide) [193]. The researchers investigated OE's ability to trigger apoptosis and/or necrosis in these cells [194] as well as the potential involvement of oxidative stress [10] and DNA injury [10]. Furthermore, the researchers discovered that both tumoral cells have identical sensitivities, but only OE had a noticeable impact on the cells. In the other side, researchers discovered that K562 cells had an increase in apoptosis, whereas Lucena cells had an increase in necrosis. Researchers discovered that OE has an antioxidant potential that protects it from oxidative harm. OE, on the other hand, caused major DNA harm on both cell lines. As a result, OE's ability to defeat the MDR phenotype means that it has anti-MDR properties [10]. In another research, the researchers investigated the cause of onion poisoning-induced hemolysis in dogs. The researchers fed cooked onions to six clinically normal and adult dogs at a pace of 30 grams per kilogram of body weight a day for two days. They took blood tests on days one, three, five, eight, twelve, eighteen, and twenty-four after onion administration, as well as urine the day after bleeding. In comparison to white blood cell counts, red blood cell volumes, hemoglobin, and hematocrit both decreased from day 1 to day 5, with a significant drop on day 5 (*P*=0.01). On days 3 (*P*=0.01) and 4, plasma bilirubin and urobilinogen levels both increased (*P*=0.01). From day one (*P*=0.01) to day three (*P*=0.01), the Heinz body counts rose marginally (*P*=0.01). The number of reticulocytes in the blood increased from day 1 to day 8, with the highest value (P 0.01) on day 8. In addition to anemia, the following erythrocyte parameters are altered: reduced nicotinamide adenine dinucleotide phosphate was reduced on day 1 (*P*=0.01); glutathione was reduced (*P*=0.01), with the lowest value on day 3 (P 0.01); glutathione peroxidase was raised on day 1, but slightly decreased on day 5 (*P*=0.01); erythrocyte membrane deformity decreased on days 1–12, while fluorescence polarization (*P*=0.01). MDA and *g* and *q* had significant correlations, with correlation coefficients of 0.922 and 0.908, respectively (*P*=0.01), but MDA and deformity index had a poor correlation, with a correlation coefficient of 0.887 (*P*=0.01). According to the experts, onion toxicity causes hemolytic anemia in puppies [11].

## 8. Clinical Studies

Several tests on human were also conducted to understand the pharmacological effect of *A. cepa* (Table 2).

## 9. Conclusion and Future Perspectives

Inflammation, cancer, diabetes, extreme wounds, gallstones, neurological disorders, and various microorganisms all respond well to onion, making it a high-value food in the therapeutics sector. Onion can be used as a natural and nontoxic substitute for a variety of nutraceutical ingredients. It owns high-food values, i.e., calcium, moisture, phosphorus, protein, iron, fat, vitamin C, minerals, carbohydrates, and fiber. Mass awareness should be created about the importance of this potential food as it has a low toxicity and only moderate side effects. In the field of pharmacology, *A. cepa* has multiaction capabilities, and researchers can continue to investigate its mode of action so that health practitioners can learn from it. Several potential prospects are there in onion research for various areas such as the creation of resistance types and hybrids to biotic and abiotic factors, higher quality standards, and antidotes for various ailments.

## Figures and Tables

**Table 1:** An overview of recent in vivo and in vitro studies of *Allium cepa* based on its therapeutic efficacy.

Field of Study	Subject	Dosage	Outcome	Mechanism of Action	References
Antimicrobial effect (in vivo)	Broiler chicks	1.5–2.5 g	Population of *E. coli* in ileum was decreased at a rate of 2.5 g/kg feed, while the amount of *Lactobacillus* was increased	Onion may alter a microflora intestinal, which improves digestion and absorption of nutrients in the intestines	[56]
Antimicrobial effect (in vitro)	*Escherichia coli* bacterial strains	Powdered bulb onion	The strain tested had MIC = 93.8 ± 44.2 µg/mL and MBC = 312.5 ± 265 µg/mL showing that *A. cepa* had antibacterial effect to a certain extent	Destroys bacteria by using their most active extract forms, or combining them to achieve latent synergistic effects	[158]
	Food-borne bacterial strains	15.6–1000 *μ*g/mL	All bacteria projected inhibition zone, but a greater inhibitory effect was observed for *S. aureus* (IZD = 6.90 ± 1.26)	n.m.	[58]
	Gram-positive and Gram-negative bacteria	n.m.	Methanolic extract of onion inhibits *E. coli* and *S. aureus.*	Flavonoids, phenolic compounds, quercetin inhibited the growth of Gram positive and Gram-negative bacteria	[45]
	Gastrointestinal tract pathogens	n.m.	100% aqueous extracts of green onion bulbs displayed maximum bacterial kill, and its kill rate is slightly higher than the kill rate by positive control for *E. aerogenes*	Flavonoids and phenolic compounds of green onion bulb destroy bacterial membrane and shows antibacterial activity	[60]
Antiplatelet activity (in vivo)	Sprague Dawley rats	6 µg/mL	Significant inhibition of aggregation of platelets	Flavones such as apigenin, chrysin, and phloretin inhibited aggregation of platelets	[87]
	Rats	5 *μ*g/mL	Platelet aggregation was inhibited	Inhibition of aggregation-inducing molecules, thromboxane A2 (TXA2), and intracellular Ca^2+^ by blocking TXA2 synthase (TXAS) and cyclooxygenase-1 (COX-1) activities	[159]
Antiplatelet activity (in vitro)	Two healthy nonsmoker donors	n.m.	The dose-response curves developed using different dosages of allium juice vs. the percentage of inhibition of aggregation are calculated for juice levels needed to reduce platelet aggregation by 50% (IC50)	Aglycone part did not take part in inhibiting platelet aggregation. The flavone part of flavonoids of *A. cepa* played the major role	[160]
Gallstone treatment (in vivo)	Sprague Dawley rats	7% (w/w) onion powder	Lowered ballooning, hepatic steatosis, and lobular inflammation	Quercetin decreases the levels of hepatic enzymes, serum lipids, steatosis, and inflammation through regulating the expressions of NF-kB, p65, Sirt1, and iNOS	[134]
Antiparasitic activity (in vitro)	*Leishmania tarentolae* and *Trypanosoma brucei brucei*	3–5 µg/mL	Zwiebelane in the onion extract killed both types of parasites efficiently	The forming of disulfide bonds between SH classes of essential redox compounds and secondary metabolites containing sulfur are inhibiting trypanothion reductases	[137]
Antidepressant activity (in vivo)	Albino Wistar mice	200 mg/kg/day	Immobilization stress substantially reduced	Reduce stress by its potential antioxidant mechanism	[144]
Anti-inflammatory (in vivo)	Wistar rats	35–140 mg/kg/day	Reduced the pulmonary inflammatory cells, such as eosinophil, neutrophil, and monocyte and overall WBC	Inhibited NF-*κ*B cells which induce inflammation	[156]
	BV-2 microglial cells	50–500 mg/mL	Attenuated neuroinflammation	Onion increases iNOS expression at the protein levels and mRNA in LPS-stimulated BV-2 microglial cells, thus reducing proinflammatory cytokines IL-1-b, TNF-a, and IL-6	[155]
	Wistar rats	150 and 300 mg/kg	Reduced lymphocyte and eosinophil count in the blood and bronchoalveolar lavage fluid (BALF)	n.m.	[154]
Inflammatory responses (in vitro)	RAW 264.7 cells	100 mg/mL	LPS-induced inflammation	Dose-dependent reduction in IL-6, TNF-a, and IL-1-b secretion, as well as NO production	[4]
Neuroprotective activity (In vivo)	Swiss albino male mice	200 mg/kg/day	Reduced lipid peroxidation and nitrate/nitrite ratios, as well as increased GSH and catalase activities. The amount of AChE in the body was also decreased.	Quercetin, kaempferol, cycloartenol, phytosterols like lophenol, 24-ethyl cycloartenol, and 24-methyl lophenol have been found to inhibit transcription of genes like FAS, S14, transferrin, apolipoprotein CIII	[161]
	Mice	300 mg/kg	Protects mice from neuronal harm in I/R induced retinal injury.	Changes the expression of neurotrophic factor	[162]
Ashthma (in vivo)	Blomia *tropicalis* (a type of mite)	100–1000 mg/kg	Induced asthma	Reduced IL-4, IL-5, IL-13, and IgE levels	[163]
Inhibitory and stimulatory activity (in vivo)	Mice	10–200 g	Inhibitory effect on Th2 activity and stimulatory effect on Th1	Th2 cytokines, IL-4, IL-5, and IL-13, as well as IgE, were inhibited at 1000 µg/mL	[164]
Osteoclastogenesis (in vitro)	RAW264.7 cells	100–1000 *μ*g/mL	Induced inflammatory conditions	Cepa inhibited the development of IL-6 and IL-1a while increasing the production of IL-3 and IL-4 and inhibiting the NF- *κ*B pathway	[165]
In breast cancer (in vivo)	Female BALB/*c* mice	0.1 mL/100 g bw	Stimulatory effects on Th1 but inhibitory effects on Th2 activity	Induced decreases in IL-4 and rises in IFN-c levels and IFN-c/IL4 ratio (Th1/Th2 balance)	[166]
Allergic rhinitis (In vivo)	BALB/c mice	20–40 mL	Decreased allergic symptoms, Reduced eosinophil penetration of nasal turbinate mucosa, and OVA-specific IgE levels	Levels of IL-4, IL-5, IL-10, IL-13, and IFN-c decreased in groups treated with onion extract	[167]
Immunomodulatory property (in vitro)	BALB/c mice	3.5–15 *μ*g/mL	Showed immunomodulatory properties	Inhibited the development of Th2 cytokines such as IL-4, IL-5, IL-13, and IgE	[164]
Immunoprotective effects (in vivo)	Wistar rats	1–100 intraperitoneal	Natural and cyclophosphamide-induced immunosuppression	TNF-a, IL-10, COX-2, IgG and IgA levels in serum were increased by and immune parameters such as myeloid cells (RBC, WBC, and hb), body weight, splenic index, and thymic index in the spleen and thymus were enhanced	[168]
Lung disorder (in vivo)	Wistar rats	0.175–0.7 mg/mL	WBC count were improved, but their lymphocytes were reduced (*p* < 0.05 to *p* < 0.001).	A significant decrease in tracheal tolerance, neutrophil and eosinophil counts, but a significant increase in lymphocyte count (*p* < 0.05 to *p* < 0.001)	[169]
Hepatoprotective (in vivo)	Adult male albino rats	200–450 mg/kg	Decreased alanine aminotransferase and overall serum bilirubin levels in a dose-dependent way	Decrease in alanine levels. Paracetamol hepatotoxic rats' aminotransferase (ALT), aspartate aminotransferase (AST), alkaline phosphatase (ALP), lactate dehydrogenase (LDH), and complete serum bilirubin (TSB).	[23]
Anti-cancer effect (in vitro)	Murine ovarian cancer model	20 mg/kg	Blocks tumor cell growth	Blocks the activation of M2 macrophages	[95]
Antidiabetic effect (in vivo)	Diabetic rats	Aqueous extract of onions (25 mg/kg) for 21 days	Reduced blood glucose level	Increased glucose uptake into soleus muscle	[101]
	Rats	200 mg/kg	Decrease in blood glucose level	Stimulate the formation of pancreatic *β* cells	[102]
	Rats	3 mL/100 g	Decrease in blood glucose level	n.m.	[100]
Antihypercholesterolemic effect (in vivo)	Sprague Dawley rats	4.5 g/kg body weight	Inhibited the formation of atherosclerosis	n.m.	[106]
	Mice	2% raw or heat processed onions with high cholesterol diet	Reduced the risk of CGS	Decrease cholesterol secretion in bile and increase bile acid excretion	[107]
Antioxidant effects (in vivo)	Broiler chicken	3 g/kg diet	Increased antioxidant enzyme activities	n.m.	[122]
Antiobesity effects (in vivo)	Rats	92.6 mg/kg bw/days	Weight gain reduced significantly compared to the rats who were only fed high fat diet	n.m.	[130]

MIC = minimum inhibitory concentration, MBC = minimum bactericidal concentration, IZD = inhibition zone diameter, n.m. = not mentioned, HDL = high density lipoprotein, LDL = low density lipoprotein, ACA = *Allium cepa* agglutinin, PROE = phenol-rich onion extract, CGS = cholesterol gallstone.

**Table 2:** Clinical studies of *Allium cepa* based on its pharmacological effects.

Field of Study	Subject	Dosage	Outcome	Mechanism of Action	References
Cardioprotective effect	24 healthy pilot	100 mL onion juice/day	Total cholesterol, waist circumference, and LDL-C reduced substantially	Onion juice contains quercetin which markedly attenuated LDL-c, serum total cholesterol, and HDL-c levels in healthy mild hypercholesterolemic subjects	[68]
Wound healing	39 patients	Onion extract (*Allium cepa*) 10%	Hypertrophic scars and keloids were attenuated properly	Onion extracts inhibit vascular endothelial growth factor (VEGF) production which is the prime cause of HTS and keloids	[82]
Antihypertensive effects	70 people	162 mg/day	Systolic BP was reduced by −3.6 mmHg	Mechanisms remain unclear	[133]
Anti-inflammatory effects	In human epidermal keratinocytes	15 mg/mL extract	Stimulatory effect on Th1 and inhibitory effect on Th2 activity	Reduced amounts of IL-6, IL-10, and TNF-*α*	[4]
Anticancer effects	Human	10 g/day	Reduced risk of prostate cancer	Activation of detoxifying enzymes by organosulfur compounds	[1]
	Human	Taking a combination of 200 mg synthetic DATS and 100 *μ*g selenium per month/year for 3 years	Gastric cancer risk is reduced	DATS triggers cancer cell cycle arrest at the G2/M phase	[96]
Antihypercholesterolemic effect	24 hypercholesterolemic patients	100 mL onion juice for 8 weeks	Reduction in serum TC, LDL-c, LDL-c/HDL-c levels	Increase LDL receptor mRNA expression and increase bile acid synthesis	[68]
Antiobesity effects	72 overweight and obese humans	50 mg	Reduction in body weight, BMI, waist circumference	As an anti-oxidant, quercetin scavenges free radicals and restores the respiration process in the adipocytes	[127,128]

BW = body weight; n.m. = not mentioned; DATS = diallyl trisulfide; TC = total cholesterol; LDL-c = low density lipoprotein cholesterol; HDL-c = high density lipoprotein cholesterol; HTS = hypertrophic scars; BP = blood pressure; OPE = onion peel extract; ZnO-NPs = zinc oxide nanoparticles.

## Data Availability

All the data are available within the article.
